# Sex-specific bi‑directional association between osteoporosis and depression from the national representative data of South Korea

**DOI:** 10.1038/s41598-022-13401-z

**Published:** 2022-06-09

**Authors:** Min Kyoung Shin, Hyejin Kim, Soo-Hee Choi, Beom-Jun Kim, Obin Kwon

**Affiliations:** 1 Department of Biomedical Sciences, Seoul National University College of Medicine, Seoul, 03080 Korea; 2 Department of Biochemistry and Molecular Biology, Seoul National University College of Medicine, Seoul, 03080 Korea; 3 Department of Public Health, Yonsei University Graduate School, Seoul, 03722 Korea; 4 Department of Psychiatry, Seoul National University College of Medicine, Seoul, 03080 Korea; 5 Department of Psychiatry, Seoul National University Hospital, Seoul, 03080 Korea; 6 Department of Endocrinology and Metabolism, Asan Medical Center, University of Ulsan College of Medicine, Seoul, 05505 Korea

**Keywords:** Endocrine system and metabolic diseases, Psychiatric disorders

## Abstract

Both osteoporosis and depression are major health threats, but their interrelationship is not clear. This study elucidated the associations between osteoporosis and depression while considering the temporal sequence of the diagnoses. In this cross-sectional study, data were extracted from the Korean National Health and Nutrition Examination Surveys (2007–2009 and 2015–2019, *n* = 29,045). Osteoporosis and depression were defined by diagnoses thereof. The odds ratio (OR) of the incident osteoporosis among depression patients without a history of osteoporosis was calculated by multivariable logistic regression adjusted for potential confounders. A reverse association was also assessed. Participants were additionally stratified by their sex and age. As a result, male depression patients aged under 50 years showed higher ORs for osteoporosis than those without depression (OR 9.16, 95% CI 1.78–47.18). Female osteoporosis patients showed lower ORs for depression than those without osteoporosis (OR 0.71, 95% CI 0.58–0.88), especially in women aged 50 years and older. In the sensitivity analysis, the same results were obtained in women by their menopause status. Depression has a strong positive association with the occurrence of osteoporosis in young male adults, and osteoporosis has a negative association with the occurrence of depression in female adults.

## Introduction

Osteoporosis is the leading endocrinological disorder that causes fractures in postmenopausal women and older men. It is a medical condition that has both social consequences and psychological ramifications for patients. One of the psychological issues that frequently emerges in people diagnosed with osteoporosis, especially with osteoporotic fracture, is depression^1^, characterized as having a depressed mood, anhedonia, and other vegetative symptoms for more than 2-week period. Both diseases place a huge burden on patients and society. Nearly 10 million Americans and above 20% of South Koreans over the age of 50 years have osteoporosis^2,3^, while more than 8% of Americans and 5% of South Koreans suffer from clinical depression^4,5^. The socioeconomic cost of osteoporosis in South Korea was recently estimated up to 982 million United States dollar (USD) in 2017, and that of depression up to totally 4 billion USD (including 169 million USD as the direct healthcare cost) in 2005^6,7^.

Much research has been performed hoping to find the association between these two diseases. Many studies have suggested that depression is correlated with a lower bone mineral density (BMD) and/or increased risk of fracture^8,9^. In cross-sectional studies and case–control studies, more studies have reported inverse associations between depression and BMD while a smaller number of studies reported no association^10^, which are largely due to heterogeneity in the design among studies and different diagnostic criteria^11^. Studies that included only one gender were common, and relatively few studies analyzed gender difference in association between depression and osteoporosis^1,12^. For the temporal association, depression was prospectively associated with a significant increase in the risk of fracture and bone loss in a recent meta-analysis of prospective studies^13^. However, whether osteoporosis is prospectively associated with changes in depression has not been studied well.

Therefore, this study investigated the bi-directional association between depression and osteoporosis, considering the temporal sequence of the diagnoses, with stratification of the sex and age groups. We used a cross-sectional evaluation of a retrospective cohort study of nationally representative sample populations (Korean National Health and Nutrition Examination Surveys; KNHANES) to find possible temporal association between depression and osteoporosis patients.

## Methods

### Subjects

In this study, we used the data from the KNHANES. Since 1998, the Korea Disease Control and Prevention Agency has conducted a repeated cross-sectional study every year to use it as a national surveillance system. The KNHANES adopted a two-stage stratified cluster sampling method based on the latest population and housing census to represent the South Korean population. Trained staff, including certified physicians, nurses, and health experts, visited selected areas and administered standardized physical examinations, health interviews, and self-reported surveys^14^. All participants provided written informed consent for the KNHANES. All procedures and protocols of the study have been approved by the institutional review board (IRB no. 2007-02CON-04-P, 2008-04EXP-01-C, 2009-01CON-03-2C, 2018-01-03-P-A, 2018-01-03-C-A) of the Korean Center for Disease Control and Prevention. The study was conducted in accordance with the Declaration of Helsinki.

**Figure 1:**
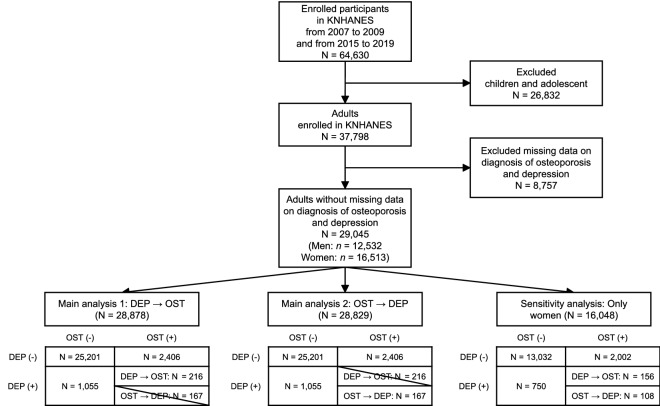
Selection of participants for analysis.

We used KNHANES data from 2007 to 2009 and 2015 to 2019 when the information about the morbidity of both depression and osteoporosis was available. During the study period, 64,630 individuals consented and enrolled in the KNHANES. For the purpose of the study, we excluded participants under 18 years of age. Additionally, we excluded participants with missing data on the diagnosis of osteoporosis or depression. Finally, 29,045 participants (45% of the total participants; 16,513 women) were included in this study (Fig. 1).

### Measurement

All the interviews and examinations in this study were conducted by trained staff using structured questionnaires and guidelines. The participants of the KNHANES reported whether they had been diagnosed with depression and/or osteoporosis by trained staff members through a structured face-to-face health interview using a structured questionnaire^14^. The clinical diagnosis was a binary variable with a “yes” or “no” response. Subsequently, if a participant responded that he/she had been diagnosed with depression and/or osteoporosis, the age at diagnosis was asked. The age at diagnosis was a continuous variable ranging from 0 to 80, and an age over 80 years was coded as 80.

Considering confounding variables when evaluating the association between depression and osteoporosis, we adjusted for several variables including age, sex, comorbid medical conditions, body mass index (BMI, kg/m^2^), smoking status, binge drinking alcohol, physical activity, education level, family income, and marital status. The age of the study participants was categorized into 19–39, 40–49, 50–59, and over 60 years. We considered additional socio-demographic variables including family income level (high, middle, and low), education level (middle school, high school, and college or higher), and marital status (with or without spouse). The other medical comorbidities were measured in the same way as depression and osteoporosis. We operationally defined participants diagnosed with diabetes, hypertension, rheumatoid arthritis, thyroid diseases, thyroid cancer, breast cancer (for women) or prostate cancer (for men) as presence of comorbid medical conditions. The BMI was calculated with body weight and height measured from the physical examination. Following the classification of BMI in adult Asians by the Western Pacific Region of the World Health Organization^15^, we divided the BMI into three categories: underweight as a BMI < 18.5, normal as 18.5 ≤ BMI < 23, and overweight/obesity as a BMI ≥ 23. Smoking status was classified into three categories including never, ever and current smoker. Additionally, binge drinking was defined by KNHANES criteria as consumption of more than 7 glasses (men) or 5 glasses (women) more than once a month in the last year. Weekly physical activity was measured based on the International Physical Activity Questionnaire (IPAQ)^16^ and dichotomized according to its presence.

### Data analysis

We considered the temporal sequence of the diagnoses of depression and osteoporosis by controlling for the age at diagnosis. We used this approach to provide rigorous evidence based on Bradford-Hill criteria beyond the limitation of cross-sectional data^17^. First, to evaluate the incidence of osteoporosis in depression patients, the individuals with a diagnosis of osteoporosis before the diagnosis of depression were excluded (Main analysis 1 in Fig. 1). In the second main analysis, we excluded participants when their diagnosis of depression preceded the diagnosis of osteoporosis (Main analysis 2 in Fig. 1). Throughout these procedures, we tried to clarify the temporal nature of the bi-directional association between depression and osteoporosis^17,18^.

Because many studies have indicated that both depression and osteoporosis show sex differences in prevalence^5,19–23^, we analyzed the data separately by sex. First, we described the characteristics of the participants with and without a diagnosis of osteoporosis by sex. Chi-squared test was conducted to test if there is a systematic difference in socio-demographic and health status between those groups. Second, we used a logistic regression model to evaluate the association between depression and osteoporosis. We estimated the odds ratio (ORs) and 95% confidence intervals (CI) of osteoporosis in patients with depression for each sex after excluding prior-osteoporosis cases,﻿ and vice versa, we also estimated the ORs and 95% CI of depression in patients with osteoporosis by sex, excluding prior-depression cases. The covariates were selected in the final model were age, comorbid medical conditions, BMI, smoking cigarettes, binge drinking alcohol, physical activity, education level, family income and marital status according to previous studies^8,10,24^. Third, we repeated the logistic regression procedures to stratify the sex (men and women) and age groups (< 50 and ≥ 50 years). Additionally, to account for the confounding effect of menopause, a sensitivity analysis was conducted only for women stratified by menopausal status^25–27^. A *p* value of < 0.05 was considered to indicate statistical significance. We used SAS 9.4 (SAS Institute Inc., Cary, NC, USA) for all statistical analyses.

## Results

Table 1 shows the baseline characteristics of the study participants according to osteoporosis in men and women. There were significant differences between the group with and without osteoporosis for both sexes in age, education, family income, marital status, smoking status, physical activity, medical comorbidity, and BMI. The difference in binge drinking was significant only in women. All the covariates that could lead to a crucial difference in the occurrence of osteoporosis were adjusted for in all the analyses of this study. The number of individuals who comorbid depression and osteoporosis were 383 during the study period, which corresponded to 26.6% of depression patients and 13.6% of osteoporosis patients.

**Table 1:** Characteristics of participants stratified by sex and diagnosis of osteoporosis using Korean National Health and Nutrition Examination Surveys (KNHANES) 2007–2009 and 2015–2019 (weighted).

Variables	Men					Women				
	With osteoporosis *n* = 179		Without osteoporosis *n* = 12,353		*p* value	With osteoporosis *n* = 2610		Without osteoporosis *n* = 13,903		*p* value
	Weighted *n*^a^ = 833,432		Weighted *n* = 97,013,943			Weighted *n* = 12,097,996		Weighted *n* = 89,528,314		
	*n*	%	*n*	%		*n*	%	*n*	%	
**Age (years)**										
18–39	7	8.24	3777	40.03	< .0001	24	1.34	4542	39.88	< .0001
40–49	5	4.83	2021	18.79		44	2.34	2666	20.01	
50–59	20	14.78	2298	19.77		349	17.11	2937	20.36	
> 60	147	72.15	4257	21.41		2193	79.20	3758	19.76	
**Education attained (years)**										
≤ 9	125	64.04	2950	17.01	< .0001	2058	75.46	3969	22.92	< .0001
10–12	28	17.39	4340	37.71		350	15.17	4616	35.74	
> 12	25	18.11	4995	44.79		184	8.60	5256	40.93	
**Family income**										
Low	86	44.79	2076	13.24	< .0001	1195	43.05	2310	14.38	< .0001
Middle	69	36.81	6419	53.45		1069	42.74	7324	53.76	
High	24	18.40	3818	33.30		329	14.21	4218	31.85	
**Marital status**										
Single	33	17.21	3351	33.17	0.0004	1080	41.22	4362	33.56	< .0001
Married	145	82.09	9002	66.83		1526	58.56	9536	66.40	
**Smoking status**										
Never	30	14.20	2905	25.49	< .0001	2421	92.64	12,176	86.66	< .0001
Past smoker	103	58.11	5172	37.39		74	2.62	900	6.71	
Current smoker	45	26.87	4221	36.78		93	3.64	763	6.22	
**Binge drinking**										
No	154	84.13	9950	79.66	0.2610	2552	97.21	13,046	93.20	< .0001
Yes	24	15.05	2351	20.00		34	1.47	796	6.41	
**Physical activity**										
Inactive	100	57.06	6428	48.90	< .0001	1566	60.41	7946	54.74	< .0001
Active	67	38.30	5845	50.54		705	28.40	5867	44.65	
**Medical comorbidity**										
No	87	48.69	8395	74.71	< .0001	1202	46.01	9834	75.35	< .0001
Yes	92	51.31	3958	25.29		1408	53.99	4069	24.65	
**Body mass index**										
Underweight	12	5.71	298	2.53	0.001	81	3.34	707	5.90	< .0001
Normal	93	50.99	5056	40.07		1282	49.49	7273	54.14	
Overweight/obesity	74	43.29	6964	56.24		1225	46.35	5752	38.48	
**Menopausal status**										
Premenopause	*NA*	*NA*	*NA*	*NA*	*NA*	55	3.17	7443	61.29	< .0001
Postmenopause	*NA*	*NA*	*NA*	*NA*		2211	85.55	6339	37.92	

Medical comorbidity was coded as ‘Yes’ if a person has one or more disease among diabetes, hypertension, rheumatoid arthritis, thyroid diseases, thyroid cancer and prostate cancer.

^a^Sum of numbers can miss the total number in group due to missing values.

*NA* not applicable.

The upper part of Table 2 shows the association between depression and osteoporosis based on the number of participants whose osteoporosis was not diagnosed before their diagnosis of depression. The total number of subjects in this part was 28,878 (12,524 men and 16,354 women), excluding 167 individuals who were diagnosed with osteoporosis preceded the diagnosis of depression. For both men and women, the prevalence of osteoporosis showed no significant difference between the participants with depression and those without depression after adjusting for all confounders in this study. The lower part of Table 2 shows the association between osteoporosis and depression based on the number of participants whose depression was not diagnosed before their diagnosis of osteoporosis. The total number of subjects was 28,829 (12,522 men and 16,307 women), excluding 216 individuals who were diagnosed with depression preceded the diagnosis of osteoporosis. For women, the prevalence of depression in those with osteoporosis was significantly lower after adjusting for all covariates (OR: 0.71; 95% CI 0.58–0.88). The prevalence of depression in men showed no difference between the groups with and without osteoporosis.

**Table 2:** Bi-directional association between depression (DEP) and osteoporosis (OST) stratified by sex.

	Group *n* (%)	Model 1		Model 2		Model 3		Model 4	
		OR	95% CI	OR	95% CI	OR	95% CI	OR	95% CI
**DEP → OST**									
**Men (*n* = 12,524)**									
Without depression	12,215 (97.53)	(ref)		(ref)		(ref)		(ref)	
With depression	309 (2.47)	2.23	(1.16–4.31)	2.07	(1.06–4.05)	1.87	(0.94–3.73)	1.86	(0.93–3.71)
**Women (*n* = 16,354)**									
Without depression	15,392 (94.12)	(ref)		(ref)		(ref)		(ref)	
With depression	962 (5.88)	1.16	(0.97–1.38)	1.12	(0.94–1.34)	0.98	(0.81–1.19)	0.99	(0.82–1.20)
**OST → DEP**									
**Men (*n* = 12,522)**									
Without osteoporosis	12,353 (98.65)	(ref)		(ref)		(ref)		(ref)	
With osteoporosis	169 (1.35)	1.75	(0.85–3.62)	1.42	(0.68–2.97)	1.49	(0.71–3.12)	1.52	(0.73–3.18)
**Women (*n* = 16,307)**									
Without osteoporosis	13,903 (85.26)	(ref)		(ref)		(ref)		(ref)	
With osteoporosis	2,404 (14.74)	0.90	(0.74–1.09)	0.83	(0.68–1.01)	0.70	(0.57–0.87)	0.71	(0.58–0.88)

Model 1: adjusted age; Model 2: Model 1 + adjusted education level, family income and marital status; Model 3: Model 2 + smoking cigarette, binge drinking alcohol and physical activity; Model 4: Model 3 + comorbid medical conditions and body mass index.

Comorbidity included diabetes, hypertension, rheumatoid arthritis, thyroid diseases, thyroid cancer, breast cancer and prostate cancer.

In the stratified analysis, likewise in the aforementioned unstratified analysis, we also adjusted for all covariates including age, comorbid medical conditions, BMI, smoking, drinking frequency, physical activity, education level, family income and marital status. Age was divided into two groups: 50 years or above and under 50 years. As shown in Table 3, when we excluded the depression patients with a prior history of osteoporosis, male participants with depression under the age of 50 years showed a significantly higher OR of 9.16 (95% CI of 1.78–47.18) for the prevalence of osteoporosis than male participants without depression, but not those older than 50 years. When we excluded osteoporosis patients with a prior history of depression, female osteoporosis patients with an age of 50 years or more showed a significantly lower OR of 0.71 (95% CI of 0.58–0.89) for the prevalence of depression than older women without osteoporosis, but not those under 50 years of age. Results of our sensitivity analysis of the menopausal status are consistent with reported findings (Table 4). Among those defined as in a postmenopausal state, the group with osteoporosis had 34% lower odds of prevalence of depression than in the group without osteoporosis.

**Table 3:** Bi-directional association between depression (DEP) and osteoporosis (OST) stratified by sex and age groups.

	Group *n* (%)	Model 1		Model 2		Model 3		Model 4	
		OR	95% CI	OR	95% CI	OR	95% CI	OR	95% CI
**DEP → OST**									
** < 50 years**									
**Men (*n* = 5810)**									
Without depression	5,688 (97.90)	(ref)		(ref)		(ref)		(ref)	
With depression	122 (2.10)	9.63	(2.09–44.48)	8.72	(1.70–44.62)	9.31	(1.83–47.36)	9.16	(1.78–47.18)
**Women (*n* = 7271)**									
Without depression	6,968 (95.83)	(ref)		(ref)		(ref)		(ref)	
With depression	303 (4.17)	1.01	(0.31–3.25)	0.78	(0.24–2.57)	0.76	(0.18–3.22)	0.73	(0.17–3.13)
** ≥ 50 years**									
**Men (*n* = 6714)**									
Without depression	6,525 (97.19)	(ref)		(ref)		(ref)		(ref)	
With depression	189 (2.81)	1.77	(0.85–3.69)	1.73	(0.82–3.63)	1.53	(0.71–3.30)	1.55	(0.72–3.33)
**Women (*n* = 9083)**									
Without depression	8,424 (92.74)	(ref)		(ref)		(ref)		(ref)	
With depression	659 (7.26)	1.27	(1.06–1.52)	1.22	(1.01–1.46)	1.07	(0.88–1.30)	1.08	(0.89–1.31)
**OST → DEP**									
** < 50 years**									
**Men (*n* = 5808)**									
Without osteoporosis	5,798 (99.83)	(ref)		(ref)		(ref)		(ref)	
With osteoporosis	10 (0.17)	*NA*^a^		*NA*		*NA*		*NA*	
**Women (*n* = 7273)**									
Without osteoporosis	7,208 (99.11)	(ref)		(ref)		(ref)		(ref)	
With osteoporosis	65 (0.89)	1.69	(0.67–4.24)	1.23	(0.47–3.19)	1.85	(0.64–5.38)	1.77	(0.61–5.15)
** ≥ 50 years**									
**Men (*n* = 6714)**									
Without osteoporosis	6,555 (97.63)	(ref)		(ref)		(ref)		(ref)	
With osteoporosis	159 (2.37)	1.75	(0.84–3.64)	1.55	(0.74–3.28)	1.67	(0.79–3.52)	1.71	(0.81–3.62)
**Women (*n* = 9034)**									
Without osteoporosis	6,695 (73.91)	(ref)		(ref)		(ref)		(ref)	
With osteoporosis	2,339 (26.09)	0.91	(0.75–1.11)	0.86	(0.71–1.05)	0.71	(0.57–0.88)	0.71	(0.58–0.89)

Model 1: adjusted age; Model 2: Model 1 + adjusted comorbid medical conditions and body mass index; Model 3: Model 2 + smoking cigarette, binge drinking alcohol and physical activity; Model 4: Model 3 + education level, family income and marital status.

Comorbidity included diabetes, hypertension, rheumatoid arthritis, thyroid diseases, thyroid cancer, breast cancer and prostate cancer.

^a^The estimates of *NA* (not applicable) were < .0001.

**Table 4:** Sensitivity analysis of association between depression (DEP) and osteoporosis (OST) in women.

	Group *n* (%)	Model 1		Model 2		Model 3		Model 4	
		OR	95% CI	OR	95% CI	OR	95% CI	OR	95% CI
**DEP → OST**									
**Premenopause (*n* = 7495)**									
Without depression	7,193 (95.97)	(ref)		(ref)		(ref)		(ref)	
With depression	302 (4.03)	0.88	(0.21–3.65)	0.78	(0.18–3.29)	0.85	(0.20–3.61)	0.83	(0.19–3.55)
**Postmenopause (*n* = 8445)**									
Without depression	7,841 (92.85)	(ref)		(ref)		(ref)		(ref)	
With depression	604 (7.15)	1.03	(0.85–1.26)	1.01	(0.82–1.23)	1.01	(0.82–1.23)	1.01	(0.82–1.23)
**OST → DEP**									
**Premenopause (*n* = 7496)**									
Without osteoporosis	7,443 (99.29)	(ref)		(ref)		(ref)		(ref)	
With osteoporosis	53 (0.71)	1.30	(0.40–4.20)	1.10	(0.33–3.63)	1.36	(0.41–4.47)	1.33	(0.40–4.40)
**Postmenopause (*n* = 8396)**									
Without osteoporosis	6,339 (75.50)	(ref)		(ref)		(ref)		(ref)	
With osteoporosis	2,057 (24.50)	0.68	(0.55–0.86)	0.66	(0.53–0.83)	0.66	(0.53–0.83)	0.66	(0.53–0.83)

Model 1: adjusted age; Model 2: Model 1 + adjusted education level, family income and marital status; Model 3: Model 2 + smoking cigarette, binge drinking alcohol and physical activity; Model 4: Model 3 + comorbid medical conditions and body mass index.

Comorbidity included diabetes, hypertension, rheumatoid arthritis, thyroid diseases, thyroid cancer, breast cancer and prostate cancer.

## Discussion

Our results show that the prevalence of osteoporosis is significantly higher in men with depression than in men without depression under 50 years of age. The prevalence of depression is significantly lower in women with osteoporosis than in women without osteoporosis, especially for those over 50 years of age when stratified. To check the robustness of our findings for the women, we performed a sensitivity analysis by redefining the effects of aging according to the menopause status in women: our results did not change; thus, this guarantees the stability of the result, at least in this population of women aged 50 or older. When examining the relationships between depression and osteoporosis, we considered the timing of the diagnosis. We investigated the sequence of the diagnoses of depression and osteoporosis to strengthen the validity of the link because causal relationships could not be verified in this study due to the cross-sectional design. The validity was additionally aided by the tight exclusion criteria, the examination of numerous confounders, and the stratification analysis.

The most prominent finding of this study was that in the osteoporosis-naïve population under 50 years of age, male participants with depression under the age of 50 years had a much higher OR of 9.16 for the prevalence of osteoporosis. Such an association was not found in female participants of any age group or in male participants with the age of 50 years or more. This finding seems to be in line with a previous report using the third National Health and Nutrition Examination Survey (NHANES III), a nationally representative sample of USA adults: in that study, both major depressive episodes and dysthymia were significantly associated with a lower BMD only in men, not in women^28^. In another prospective cohort study of community-dwelling older men of 50 years of age or older, depressive symptoms were neither associated with BMD nor changes in BMD per year^12^. In our study, association between depression and incident osteoporosis was not evident when all the participants were combined, which emphasizes the importance of stratification during data analysis. Several studies have found a link between depression and a low BMD, although inconsistent results have made it difficult to draw a definite conclusion. In a longitudinal study, Schweiger et al. found evidence of greater bone density loss in the depressed group of men and women compared to the controls after two years of follow-up^29^. In another population-based cohort study, however, there was no link identified between the mean BMD and the number of times women and men reported depression^30^. The selection of the study population and methods of measuring exposure were heterogeneous among the studies, which might contribute to the inconsistency of the association.

We speculated on the mechanism why this association is only significant in the young male group. Both biological and behavioral mechanisms may have a role in the relationship between depression and incident osteoporosis^10^. For behavioral mechanisms, more common are smoking, less physical activity, and alcohol abuse in depressed people. These effects have already been adjusted for in the analysis, so they may not be possible to directly explain this association. Biological mechanisms include high levels of cortisol, sympathetic activation, and low levels of gonadal steroids. The sex-different effect of hypercortisolism on BMD can be one of the candidates to explain our results. In Cushing’s disease patients, men have presented a higher prevalence of osteoporosis compared to women^31^. In another recent cohort study for Cushing’s syndrome, men showed a higher prevalence of osteoporosis with more vertebral fractures than women^32^. Although the degree of hypercortisolism of Cushing patients are different from that of depression patients, it is plausible to hypothesize that men might be more susceptible to stress-induced bone mass loss. For sympathetic activation and related catecholamine metabolism, depressed men showed a greater sympatho-vagal activity than depressed women in a young adult group^33^. In a humanized mice study, activation of the renin-angiotensin system induced osteoporosis only in the male group^34^, although more studies are needed to validate the result in humans. Another candidate can be neuropeptide Y (NPY), a polypeptide that has the osteogenic ability to proliferate and differentiate bone marrow stem cells^35^. Patients with depression had lower levels of NPY in plasma compared to controls, whereas female patients had significantly higher levels of NPY than male patients^36^. Considering the anti-depressive potential of NPY^37,38^, NPY may be suggested as one of the protective mechanisms from osteoporosis in women with depression. Indeed, from a clinical aspect, women were more likely than men to achieve remission from depressive disorders in Korea that spanned similar time periods to ours^39^. In that analysis, there were no significant differences in treatment-related parameters such as antidepressant regimen between male and female patients. Still, the following are some probable explanations for the gender disparities in the antidepressant treatment response: (1) serotonergic potency of selective serotonin reuptake inhibitors (SSRIs) has been suggested as more important for women, especially at a younger age^40^; (2) estrogen may have an effect in determining the antidepressant response with enhancing serotonergic activity^41^. However, at older ages, all the effects above can be masked by multiple factors contributing to bone mass loss, such as age-related decline in sex hormone levels, nutritional disturbances, changed responsiveness of hypothalamic–pituitary–adrenal (HPA) axis, and multiple drug treatments^42,43^.

An unexpected finding in this study was that in the depression-naïve population over 50 years of age, aged female participants with osteoporosis had a significantly low OR for the prevalence of depression. Such an association was not found in male participants of any age or in female participants under the age of 50 years. This analysis is further strengthened by the same result from the sensitivity analysis according to menopausal status in the female group. This result is contrary to the impression that osteoporosis might negatively affect mood due to impaired physical ability and reduced quality of life^44^. However, there is no substantial evidence that osteoporosis itself causes psychiatric problems, and no indication that it can induce depression. Moreover, not a low BMD but related fractures seem to be associated with the incident depression^1^.

Cumulative evidence has shown the effect of bone-derived factors on brain function^45^. One of the putative factors relevant to our result can be osteocalcin, a bone matrix protein released from osteoblasts into the circulation in an undercarboxylated form. Interestingly, osteocalcin can have an anti-depressive effect. After crossing the blood–brain barrier (BBB), bone-derived osteocalcin can bind to serotonergic neurons. Mice lacking osteocalcin show a substantial increase in depression-like behavior compared to wild-type littermates, which was corrected by intracerebroventricular infusion of osteocalcin^46^. When facing acute danger, osteocalcin may be necessary to develop an acute stress response through inhibition of parasympathetic tone^47^. In postmenopausal women, the osteocalcin levels show a positive correlation with age^48^ and negative correlation with the BMD^49^. Additionally, in a Korean population, women aged 20–70 years had undercarboxylated osteocalcin inversely related to BMD independent of other factors that may influence the BMD^50^. These population-based data of osteocalcin levels might be related to a low risk of depression in aged women with osteoporosis. In the male population, on the other hand, there is only limited evidence for correlation between osteocalcin and BMD in alcoholic patients^51^. Other bone-derived factors include osteoblast-derived lipocalin 2 (LCN2). LCN2 can cross the BBB^52^, and the serum LCN2 level is elevated in older osteoporotic women compared to a younger population^53^. LCN2-null mice exhibit depressive-like behaviors^54^, while LCN2 expression in the hippocampus can rescue this phenotype^55^. All these bone-derived factors can be candidates to mediate a lower prevalence of depression in osteoporosis women, and further validation in the human population is needed.

Medications for osteoporosis may have an effect on the low risk of depression in older women with osteoporosis. Hormone replacement therapy (HRT) containing estrogen, prescribed only for perimenopausal osteoporosis patients, has an anti-depressive effect^56^. Tibolone, a selective tissue estrogenic activity regulator usually prescribed for HRT candidates, also significantly improves depressive symptom^57^. Raloxifene, a selective estrogen receptor modulator, could alleviate depressive symptom in osteopenic postmenopausal women in a randomized controlled trial^58^. Zoledronate, a kind of bisphosphonate, showed an anti-depressive effect in postmenopausal women^59^, and alendronate ameliorated depressive-like behavior in a menopausal experimental model^60^. All these medications were recommended in the Korean treatment guideline used in the same period when the dataset of this study was collected^61^ and might be partially involved in the anti-depressant effect on aged women with osteoporosis.

Although the 95% CI of the decreased adjusted OR (0.58–0.89, see Table 3) of depression among women with osteoporosis 50 years or older is narrow, we cannot rule out the possibility of bias due to unmeasured confounders. Accessibility to mental health services, due to physical malfunction, can be different between women with and without osteoporosis, which can lead to lower odds of a diagnosis of depression^62,63^. Moreover, the existing symptoms of depression can be masked by the diagnosis of physical illnesses such as osteoporosis because the diagnosis of depression requires the exclusion of direct effects of other medical conditions such as osteoporosis or menopause^64,65^.

The main strength of the present study is that we analyzed a bi-directional association between two diseases considering the temporal sequence of the diagnoses: by excluding patients who already had disease A from the denominator, the risk that a person with disease A would later have another disease B (or vice versa) could be calculated. This method is useful in that the exposure must precede outcome. We also could solidify one of the main results by sensitivity analysis: it remained consistent even after stratification by either the women’s age or menopause status, both of which are potential factors that may have a significant impact on the study’s outcome in terms of female sex hormone physiology.

Although our study was based on nationally representative samples from Korea, there are several limitations. KNHANES was designed as a repeated cross-sectional study, and a longitudinal follow-up for each participant is impossible. Data on some disease conditions were not analyzed by cross-referencing relevant laboratory data such as dual-energy X-ray absorptiometry (DXA) findings for the diagnosis of osteoporosis; thus, the possibility of false negatives cannot be completely ruled out. Fractures due to osteoporosis were not included in the dataset or in the analysis, although fractures and the related immobility per se can be more stressful to patients. Finally, some of the observed numbers, for example, males younger than 50 years of age with depression, were too small for the statistical power to be sufficient. However, because the lowest CI of the adjusted OR for osteoporosis diagnosis is 1.78 for the younger men with depression compared to younger men without depression, the estimate still implies a significant finding. A well-designed prospective cohort study with a large sample size may overcome these limitations in the future.

In conclusion, by analyzing a nationwide population-based cohort study, we discovered that depression has a strong positive association with the occurrence of osteoporosis in young male adults, while osteoporosis has a negative association with the occurrence of depression in old female adults. Several biological mechanisms may be relevant to these results, which need further research to confirm them.

## Data Availability

The Korea Centers for Disease Control and Prevention (KCDC) has supported researchers in Korea by providing annual workshops for data users. The KCDC has published the Korea Health Statistics each year, and microdata are publicly available through the KNHANES website (http://knhanes.cdc.go.kr).
